# Diagnosis of lung cancer in individuals with solitary pulmonary nodules by plasma microRNA biomarkers

**DOI:** 10.1186/1471-2407-11-374

**Published:** 2011-08-24

**Authors:** Jun Shen, Ziling Liu, Nevins W Todd, Howard Zhang, Jipei Liao, Lei Yu, Maria A Guarnera, Ruiyun Li, Ling Cai, Min Zhan, Feng Jiang

**Affiliations:** 1 Department of Pathology, University of Maryland School of Medicine, 10 S. Pine St. Baltimore, MD 21201, USA; 2 Department of Oncology, The First Hospital of Jilin University, 1 Xinmin St. Changchun, Jilin 130021, China; 3 Department of Medicine, University of Maryland School of Medicine, 22 S. Greene St. Baltimore, MD 21201, USA; 4 Department of Surgery, University of Maryland School of Medicine, 22 S. Greene St. Baltimore, MD 21201, USA; 5 Department of Epidemiology & Public Health, University of Maryland School of Medicine, 660 W. Redwood St. Baltimore, MD 21201, USA

## Abstract

**Background:**

Making a definitive preoperative diagnosis of solitary pulmonary nodules (SPNs) found by CT has been a clinical challenge. We previously demonstrated that microRNAs (miRNAs) could be used as biomarkers for lung cancer diagnosis. Here we investigate whether plasma microRNAs are useful in identifying lung cancer among individuals with CT-detected SPNs.

**Methods:**

By using quantitative reverse transcriptase PCR analysis, we first determine plasma expressions of five miRNAs in a training set of 32 patients with malignant SPNs, 33 subjects with benign SPNs, and 29 healthy smokers to define a panel of miRNAs that has high diagnostic efficiency for lung cancer. We then validate the miRNA panel in a testing set of 76 patients with malignant SPNs and 80 patients with benign SPNs.

**Results:**

In the training set, miR-21 and miR-210 display higher plasma expression levels, whereas miR-486-5p has lower expression level in patients with malignant SPNs, as compared to subjects with benign SPNs and healthy controls (all P ≤ 0.001). A logistic regression model with the best prediction was built on the basis of miR-21, miR-210, and miR-486-5p. The three miRNAs used in combination produced the area under receiver operating characteristic curve at 0.86 in distinguishing lung tumors from benign SPNs with 75.00% sensitivity and 84.95% specificity. Validation of the miRNA panel in the testing set confirms their diagnostic value that yields significant improvement over any single one.

**Conclusions:**

The plasma miRNAs provide potential circulating biomarkers for noninvasively diagnosing lung cancer among individuals with SPNs, and could be further evaluated in clinical trials.

## Background

Lung cancer is the second most common cancer and the number one cancer killer in the USA and worldwide [1]. Lung cancer is often diagnosed at an advanced stage. The 5-year survival rate for stage IV lung cancer is only 10%, whereas approximately 80% for stage IA disease [2,3]. These statistics provide the primary rationale to improve lung cancer screening and early detection [4]. Chest X-ray and sputum cytology have been used for lung cancer screening. However, the sensitivity was low [2,3]. Several randomized trials in the USA and Europe have been carried out with the hope that high-resolution CT imaging can detect lung cancer earlier, much as screening has done for breast and colorectal cancer [5,6]. Recently, the National Lung Screening Trial sponsored by the National Cancer Institute determines that CT scan offers a lung cancer-specific mortality reduction of 20.3% compared with X-ray in people who are at high risk to develop lung cancer [5-8]. Given the continued efforts to search for proof that lung cancer early detection can improve outcomes of the malignancy, this is certainly an exciting and encouraging finding. However, the widespread use and improved sensitivity of CT dramatically increase the number of solitary pulmonary nodules (SPNs) seen in asymptomatic individuals. Yet only a small fraction of SPNs are lung tumors [9-12]. Therefore, it is imperative to make a definitive preoperative diagnosis of SPNs so that lung cancer can be found in the earliest, most curable stage, while sparing benign growths from invasive biopsies and treatments [7,10-12].

The Fleischner Society [7,9] proposes a work-up for managements of SPNs, which includes surgical resection, transthoracic needle biopsy, and observation with serial chest radiographs. Each approach has advantages and disadvantages [9-12]. Surgery is the diagnostic criterion standard and definitive treatment for malignant SPNs, but should be avoided in cases of benign growths. Needle biopsy can establish a specific benign or malignant diagnosis, but is invasive, potentially risky, and sometimes nondiagnostic [9]. Observation with serial chest radiographs avoids unnecessary surgery in cases of benign disease but delays appropriate diagnosis and treatment, when malignancy is really existent [8]. Furthermore, the non-surgical approaches may lead to unnecessary radiation exposure, procedures, anxiety, cost, and low accuracy [9,10]. Therefore, it is clinically important to develop new techniques for noninvasively diagnosing lung cancer with high accuracy. One approach is to identify lung cancer-associated molecular genetic changes in biological fluids, and hence develop noninvasive and cost-effective biomarkers. Blood is an easily accessible and rich body fluid. Furthermore, blood plasma contains cell-free DNA and RNA that provide potential circulating biomarkers [13]. A handful of lung cancer-related molecular genetic abnormalities have been identified in last decades [13,14]. Yet none has provided sufficient evidence to be clinically useful for lung cancer diagnosis [15].

MicroRNAs (miRNAs) play important functions in diverse biological processes, including development, cell proliferation, differentiation, and apoptosis [16]. miRNAs can transcriptionally regulate expressions of more than 30% of human protein coding genes [16,17]. Some miRNAs act as oncogenes or tumor suppressors in tumorigenesis [17]. Therefore, the altered miRNA expressions can contribute to the development and progression of tumorigenesis [18]. Furthermore, specific over- or under-expression of some miRNAs correlate with particular tumor types, and thus open up a new field for molecular diagnosis of cancer [19-22]. For instance, measuring expression levels of a single miRNA, miR-205, in surgically resected lung tumor tissues can identify squamous cell carcinoma of the lungs with 96% sensitivity and 90% specificity [22]. In addition, measuring blood-based miRNA expressions can be used to predict survival of lung cancer patients [23-25]. Therefore, plasma miRNAs could function as circulating biomarkers. We recently showed that aberrant plasma expressions of miRNAs could distinguish lung cancer patients from healthy individuals [26-29].

The objective of the current study was to investigate whether the plasma miRNAs have the potential to be used as biomarkers in identification of lung cancer among individuals with CT-detected SPNs. Our results show that combined use of three miRNAs might provide circulating biomarkers for distinguishing lung tumors from benign SPNs.

## Methods

### Patients and clinical specimens

Sixty-five patients with SPNs were used as a training set for discovery of miRNA biomarkers. The patients consisted of 32 individuals who had malignant SPNs and 33 subjects with benign SPNs. Twenty nine healthy smokers without SPN were also recruited as controls. The demographic and clinical variables of the cases and controls are shown in Table 1. Furthermore, an independent group of 156 patients who had SPNs was used as a testing set for validation of the miRNA biomarker. The testing set consisted of 76 patients with malignant SPNs and 80 patients with benign SPNs, whose demographic and clinical variables are shown in Table 2. All patients were selected based on presence of SPNs on chest CT scan. Final diagnoses were confirmed with histopathologic examinations of specimens obtained by CT-guided transthoracic needle biopsy, transbronchial biopsy, videotape-assisted thoracoscopic surgery, or surgical resection. The study was approved by appropriate institutional review boards. Ten ml of peripheral blood was drawn from the subjects using standardized phlebotomy procedures in BD Vacutainer spray-coated K2EDTA Tubes (BD, Franklin Lakes, NJ). The blood samples from cancer patients were collected prior to definitive surgery, administration of anesthesia, and adjuvant therapy. The specimens were processed within 2 hours of collection by centrifugation at 1,300 × G for 10 minutes at 4°C. Plasma was transferred to a fresh tube and stored at -80°C until use.

**Table 1:** The demographic and clinical variables of a training set of patients with malignant SPNs, patients with benign SPNs, and healthy smokers

	32 Patients with malignant SPNs	33 Patients with benign SPNs	29 Healthy controls
Age, Median (SD)	66.2 (8.3)	64.7 (12.9)	66.5 (7.1)
Sex			
Female	12 (37.5%)	13(39.4%)	10 (34.5%)
Male	20 (62.5%)	20 (60.6%)	19 (65.5%)
Race			
African American	8 (25.0%)	9 (27.3%)	8 (27.6%)
White	24 (75.0%)	24 (72.7%)	21 (72.4%)
Nodule size, Median (SD)	2.6 (1.8)	1.1 (1.7)	
Pack-years, Median (SD)	35.5 (26.8)	30.0 (30.2)	35.0 (22.8)
Stages of NSCLC			
stage I	11 (34.4%)		
stage II	8 (25.0%)		
stage III-IV	13 (40.6%)		
Histological types of NSCLC			
AC	18 (56.3%)		
SCC	14 (43.7%)		
Diagnosis of benign SPNs			
Inflammatory lesion		27 (81.8%)	
Sarcoid		3 (9.1%)	
Granuloma		2 (6.1%)	
Fibrosis		1 (3.0%)	

Abbreviations: SPNs, solitary pulmonary nodules; SD, standard deviation; NSCLC, non-small-cell lung cancer; AC, adenocarcinoma; SCC, squamous cell carcinoma

**Table 2:** The demographic and clinical variables of a testing set of patients with malignant SPNs and patients with benign SPNs

	76 patients with malignant SPNs	80 patients with benign SPNs
Age, Median (SD)	67.9 (7.6)	65.4 (13.7)
Sex		
Female	34 (44.7%)	30 (37.5%)
Male	42 (55.3%)	50 (62.5%)
Nodule size, Median (SD)	2.5 (1.7)	1.0 (1.4)
Pack-years, Median (SD)	35.5 (23.7)	35.0 (26.7)
Stages of NSCLC		
stage I	24 (31.6%)	
stage II	30 (39.5%)	
stage III-IV	22 (28.9%)	
Histological types of NSCLC		
AC	40 (52.6%)	
SCC	36 (47.4%)	
Diagnosis of benign SPNs		
Inflammatory lesion		60 (75.0%)
Sarcoid		8 (10.0%)
Granuloma		7 (8.8%)
Fibrosis		5 (6.2%)

Abbreviations: SPNs, solitary pulmonary nodules; SD, standard deviation; NSCLC, non-small-cell lung cancer; AC, adenocarcinoma; SCC, squamous cell carcinoma.

### RNA isolation

RNA was extracted from plasma by using a mirVana miRNA Isolation Kit (Ambion, Austin, TX) as described in our previous study [25-28]. Purity and concentration of RNA were determined by using a dual beam UV spectrophotometer (Eppendorf AG, Hamburg, Germany). Integrity of RNA was determined by using a Bioanalyzer 2100 (Agilent Technologies, Santa Clara, CA). RNA samples with an RNA integrity number > 8 underwent in further analysis.

### Quantitative reverse transcriptase PCR (qRT-PCR)

qRT-PCR was carried out with TaqMan MicroRNA RT Kit (Applied Biosystems, Foster City, CA) as described in our published works [26-29]. Expression levels of five miRNAs in plasma were calculated using comparative cycle threshold (Ct) method with the equation 2-ΔΔCt. The five miRNAs included miRs-21, 126, 210, 375, and 486-5p. Ct values of the target miRNAs were normalized in relation to that of miR-16. MiR-16 was proven as one of commonly used internal control genes to determine expressions of miRNAs as showed in our and others' previous work [29,30].

### Statistical analysis

To determine sample size, we used the area under receiver operating characteristic curve (AUC) analysis and set the null hypothesis (H0) at 0.5. Accordingly, at least 28 subjects were required in each category of cases and controls to show a minimum difference of interest between an AUC of 0.75 versus an AUC of 0.5 with 80% power at the 5% significance level [31,32]. Therefore, the training and testing sets of cohorts (Tables 1 and 2) enrolled in the present study provided an enough statistical power for identification and verification of the biomarkers. Pearson's correlation analysis was applied to assess relationship between plasma miRNA expressions and demographic and clinical characteristics of the patients and control individuals. The receiver-operator characteristic (ROC) curve and AUC analyses were used to determine sensitivity, specificity, and corresponding cut-off value of each miRNA [31,32]. To decide sensitivity and specificity, clinicopathologic results were used as the reference standards. Logistic regression was used to develop composite panels of biomarkers to identify a panel that could distinguish malignant from benign SPNs with the highest sensitivity and specificity. All analyses including correlation coefficient, Wilcoxon test, logistic regression, ANOVA, and t test were performed using log transformed data. All P values shown were two sided, and a P value of < 0.05 was considered statistically significant. Graphical displays were prepared by using GraphPad Software (GraphPad Software, Inc, La Jolla, CA) to show the distributions of plasma expression for each miRNA in each group.

## Results

### Identification of miRNAs whose aberrant plasma expressions are associated with malignant SPNs

**Figure 1:**
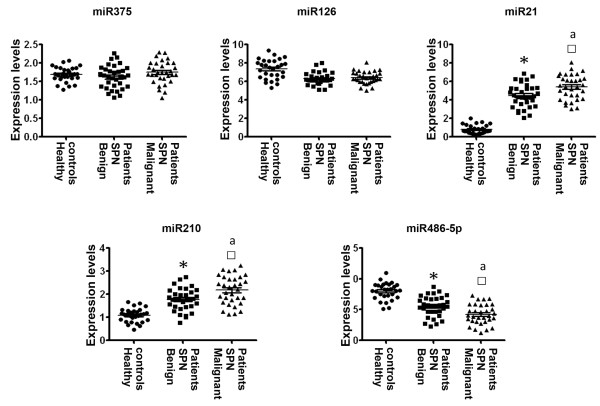
**Comparison of plasma miRNA expressions in healthy smokers, patients with benign SPNs, and patients with malignant SPNs**. Horizontal lines indicate mean values. *, statistical significance (P < 0.05) between patients with benign SPNs and healthy controls. ^□^, statistical significance (P < 0.05) between patients with malignant SPNs and healthy controls. ª, statistical significance (P < 0.05) between patients with malignant SPNs and patients with benign SPNs.

Based on our previous work using surgical tissues [26-29], five miRNAs (miRs-21, 126, 210, 375, and 486-5p) were selected in this present study. Our previous studies [26-29] showed that miRs-21, 210, and 375 had higher expression levels, whereas miRs-126 and 486-5p displayed lower expression levels in lung tumors compared with normal lung tissues. In the current study, all miRNAs tested had ≤ 30 Ct values in each plasma sample. The results indicated that the miRNAs stably existed in plasma and could be robustly detectable through qRT-PCR assay. Plasma expression level for each miRNA was further compared between three groups of subjects in the training set. As shown in Table 3 and Figure 1, both miR-126 and miR-375 did not show statistical difference between the three groups (All P > 0.100). miR-21 and miR-210 displayed higher plasma expression levels in patients bearing benign SPNs compared to healthy smokers (All P ≤ 0.001). Furthermore, their plasma expression levels were even higher in patients with malignant SPNs compared to individuals with benign SPNs (All P < 0.010). In contrast, plasma miR-486-5p expression was reduced in patients with benign SPNs compared to healthy smokers (P < 0.0001). Furthermore, plasma mir-486-5p expression level in patients having malignant SPNs was even lower compared to patients with benign SPNs (P = 0.0048) (Table 3 and Figure 1). Taken together, miR-21 and miR-210 displayed significantly higher plasma expression levels in patients with malignant SPNs compared to both subjects having benign SPNs and healthy smokers (Figure 1). miR-486-5p had considerably lower plasma expression level in patients bearing malignant SPNs compared to both subjects having benign SPNs and healthy smokers (Figure 1). Therefore, the three miRNAs present potential plasma biomarkers for identifying malignant SPNs.

**Table 3:** Expression levels of plasma miRNAs in healthy smokers, patients with benign SPNs, and patients with malignant SPNs

	Healthy smokers	Patients with benign SPNs	Patients with malignant SPNs
	
MiRNAs	Expression levels of each miRNA, Median (SD)		
miR-375	1.698 (0.1996)	1.611 (0.3110)	1.735 (0.3027)
miR-126	7.531 (1.050)	6.133 (0.6545)	6.292 (0.6652)
miR-21	0.639 (0.4707)	4.401 (1.2820)^□^	5.47 (1.3450)*
miR-210	1.126 (0.2952)	1.769 (0.4759)^□^	2.252 (0.6174)*
miR-486-5p	8.088 (1.3610)	5.668 (1.641)^□^	4.019 (1.6290)*

SPNs, solitary pulmonary nodules; SD, standard deviation. *, statistical significance between patients with malignant SPNS and patients with benign SPNs. □, statistical significance between patients with benign SPNs and healthy controls.

ROC analyses were performed to evaluate the capability of using the three miRNAs (miRs-21, 210, and 486-5p) to discriminate patients with malignant SPNs from patients having benign SPNs. The individual miRNAs exhibited AUC values of 0.5767-0.6913 (Additional file 1, Table S1; Additional file 2, Figure S1). As a result, they yielded 56.25-71.80% sensitivity and 57.58-72.73% specificity in distinguishing malignant from benign SPNs. Furthermore, using Logistic regression analysis, we identified a linear combination of values for plasma miRs-21, 210, and 486-5p expression levels. The three miRNAs used in combination could create a model to distinguish malignant from benign SPNs (Table 4). When optimum cut-offs were selected for the three miRNAs at 5.3941, 2.0008, and 5.0215, respectively, their combined use generated 0.8551 AUC, which was significantly higher than that of any single gene alone (P < 0.05). Accordingly, the composite panel of the three miRNAs revealed 75.00% sensitivity and 84.85% specificity in diagnosis of lung cancer from CT-detected SPNs. The parameters were significantly higher compared with that of individual miRNAs (all p < 0.05). Taken together, combined analysis of the three miRNAs had a reasonable power to identify lung tumors in patients with SPNs.

**Table 4:** Panels of biomarkers that differentiate malignant from benign SPNs determined by Logistic regression analysis

		Mann-Whitney		
	
ROC model	Area	Standard error	95% Wald confidence limits	
miR-21 and miR-210	0.775	0.0582	0.6606	0.8886
miR-21 and miR-486-5p	0.773	0.0581	0.6588	0.8867
miR-210 and miR-486-5p	0.814	0.0527	0.7111	0.9177
miR-21, miR-210, and miR-486-5p	0.855	0.0456	0.7657	0.9445

ROC, the receiver-operator characteristic curve; AUC, the area under receiver operating characteristic curve.

Pearson correlation analysis indicated that the estimated correlations among expression levels of the three miRNAs in plasma were low (All P > 0.05) (Additional file 3, Table S2). The data suggested that expressions of the miRNAs were complementary to each other, and further supported that combing the three genes outperformed a single one used alone. Furthermore, none of the three miRNAs had statistical differences in term of diagnostic sensitivity and specificity between different stages of lung tumors (P > 0.05) (Additional file 4, Table S3). In addition, there was no association of the changes of the genes with the age, gender, ethnicity, and histological type of the participants (All p > 0.05), but their smoking pack-years (p < 0.05) (Additional file 4, Table S3). Moreover, plasma expression levels of the miRNAs were related to size of SPNs (p < 0.05) (Additional file 4, Table S3).

### Validating the identified miRNAs in a testing set of cases and controls

To further evaluate the diagnostic performance of the small miRNA panel, the three miRNAs were assessed in a testing cohort (Table 2). miR-21 and miR-210 displayed higher plasma expression levels in patients with malignant SPNs compared to individuals having benign SPNs (All p < 0.05). In contrast, miR-486-5p had lower expression level in patients with malignant SPNs compared to individuals having benign SPNs (P < 0.01). The observations were in agreement with the findings observed in the above training test. Furthermore, we used the optimal thresholds established in the above training set to determine diagnostic performance of the miRNAs in the testing set. The panel of the three miRNAs produced 76.32% sensitivity and 85.00% specificity in differentiating malignant from benign SPNs (Table 5). The three miRNAs had no statistical differences of diagnostic sensitivity and specificity between stages of lung tumors (P > 0.05) (Table 5). Furthermore, the changes of the genes had no association with the age, gender, and histological type of the participants (All p > 0.05), except their smoking pack-years (p < 0.05) (Additional file 5, Table S4). Finally, plasma expressions of the miRNAs were correlated to size of SPNs (p < 0.05) (Additional file 5, Table S4). Altogether, the validation data created from the testing set confirmed that the miRNAs had the potential to be used as biomarkers for lung tumors among individuals with CT-detected SPNs.

**Table 5:** Diagnostic performance of the composite panel of three miRNAs in a testing cohort of patients with malignant SPNs and patients with benign SPNs

	Sensitivity	Specificity
All cases	76.32%	85.00%
Different histological types *		
AC	75.00%	85.00%
SCC	77.78%	85.00%
Cases with different stages *		
I	75.00%	85.00%
II	80.00%	85.00%
III-IV	72.73%	85.00%

Abbreviations: SPNs, solitary pulmonary nodules; SCC, squamous cell carcinoma; AC, adenocarcinoma. *P > 0.05.

## Discussion

In the present study, we investigate whether plasma miRNAs could be useful in distinguishing malignant from benign SPNs discovered by CT scan. We first determine plasma expression levels of five miRNAs in a training set of cases and controls. We find that three miRNAs used in combination has higher diagnostic efficiency for lung cancer than does any single one. Furthermore, the validation of the miRNA panel in a testing set confirms that the three genes could provide biomarkers for lung cancer diagnosis in subjects with SPNs.

As an oncomir, up-regulation of miR-21 leads to tumor development and progression [33-35]. Circulating miR-21 has been described as a biomarker for different tumor entities [36,37]. For instance, we recently found that miR-21 was one of plasma miRNAs that could differentiate early stage lung cancer patients from healthy non-smoking individuals [29]. miR-486-5p was shown to regulate tumor progression and OLFM4 anti-apoptotic factor [38]. Furthermore, miR-486-5p was underexpressed in several types of solid tumors [25,39,40]. We also found that miR-486-5p displayed lower expression levels in lung tumor tissues compared with the paired normal lung tissues [26,29]. In line with the previous findings in tissue specimens, the current study shows that plasma miR-486-5p expression in lung cancer patients is significantly lower compared to subjects with both benign SPNs and healthy smokers. The findings in both surgical tissues and plasma specimens suggest that miR-486-5p down-regulation might play a role as a tumor suppressor in lung tumorigeneisis. We are pursuing a study to investigate possible mechanism of down-regulation of miR-486-5p in the development and progress of lung cancer. miR-210 regulates the hypoxic response of tumor cells and tumor growth [41]. Overexpression of miR-210 is related to aggressiveness of breast and oral cancers [42]. Furthermore, up-regulation of miR-210 can mediate mitochondrial alterations associated with modulation of HIF-1 activity in late stages of lung cancer [43]. In addition, elevated miR-210 expressions in serum could be used as one of biomarkers for diffuse large B-cell lymphoma and pancreatic cancer [44,45]. We recently reported that miR-210 might be a plasma biomarker for diagnosis of early stage non-small-cell lung cancer [29]. In the present study, we extend our previous findings by demonstrating that the miRs-21, 210, and 486-5p have the potential to be used as circulating biomarkers for distinguishing malignant from benign SPNs detected by CT.

Major causes of benign SPNs include chronic smoking, granuloma, and infections, et al [9,12]. These benign diseases could exhibit some molecular changes [4,46-48]. Therefore, it is not surprise to find that individuals with benign SPNs have higher degrees of aberrant plasma expressions of miRNAs compared with healthy individuals. However, lung cancer patients have even higher degrees of abnormal plasma expressions of the miRNAs compared with subjects with benign SPNs. Taken together, lung cancer patients have higher degrees of abnormal plasma changes for the three miRNAs compared to both individuals and healthy smokers. Therefore, the miRNA biomarkers could distinguish lung cancer patients from subjects bearing benign SPNs and healthy smokers.

Analysis of cancer-associated miRNAs in blood for lung cancer diagnosis has been investigated by several laboratories [29,49-51]. For instance, Boeri et al recently showed that specific miRNA signatures in plasma could be useful for lung cancer diagnosis and identifying the onset of aggressive disease even before spiral-CT detection [50]. The finding from our current research supports the previous observations. Moreover, the sensitivity and specificity of combined use of the three plasma miRNAs for identifying malignant SPNs is higher compared with individual genes. Given the accuracy, future combined use of the miRNAs with CT will overcome the weakness of the imaging analysis that has low sensitivity in the early detection of lung cancer. Such combination will also surmount major obstacle of circulating biomarkers, which cannot localize tumor location. This concept is supported by our recent study in which, we demonstrated that combined use of sputum-based genomic probes with CT increased the diagnostic efficiency for stage I lung cancer compared with CT used alone [52]. Therefore, the plasma miRNAs would be an aid to decision making in the management of lung SPNs. In addition, it has been believed that malignant lesions of the lungs are more common in bigger SPNs [9]. Because the identified miRNAs whose aberrations are malignancy-related changes, it is not surprise to find that there is statistical correlation between expression levels of the genes with size of SPNs. Moreover, none of the circulating miRNAs shows correlation to histological subtypes. Interestingly, altered expressions of the miRNAs are found not only in advanced stage, but also in early stage lung cancers. The discovery might be an important characteristic if the miRNAs are to be employed for the early detection of the malignancy.

Our result appears promising, because compared with current techniques, the assay is less expensive and a noninvasive approach, thus offering a potential cost-effective approach in discriminating malignant from benign SPNs. However, the miRNAs biomarkers have several limitations. First, the panels of biomarkers were selected from a limited numbers of miRNA candidates. Other important miRNAs might not be included. Therefore, the accuracy of the miRNA-based biomarkers for identifying malignant SPNs is not high enough to be used in clinical settings. To overcome the obstacle, we are applying comprehensive miRNA microarrays to analyze plasma miRNA profile of patients with malignant SPNs, and comparing to those in patients with benign SPNs. The globally search for genome-wide miRNA profiling will identify additional more informative miRNAs for malignant SPNs. The important miRNAs can be added to the current ones to improve the diagnostic efficacy of the plasma miRNA biomarkers. Second, malignant SPNs are highly heterogeneous: most of malignant SPNs are primary lung tumors, and some are metastases from other organs. Whether the three miRNAs are useful in detection of metastatic cancer in the lungs remains to be investigated.

## Conclusions

We report the identification and evaluation of a small panel of miRNAs that provides potential biomarkers for diagnosis of lung cancer in subjects with CT-found SPNs. Nevertheless, undertaking a multicenter clinical trial in a large population to prospectively validate our current findings is certainly needed. After the comprehensive and definite validation, future use of the biomarkers would complement CT in lung cancer screening for the early detection of lung cancer.

## Competing interests

The authors declare that they have no competing interests.

## Authors' contributions

JS, ZL, JL, LY and RL conducted the experiments and participated in data interpretation. NWT, HZ and MAG participated in study design, coordination, and acquisition of data. LC and MZ participated in data analysis. FJ participated in study design, coordination, and data analysis and interpretation, and prepared the manuscript. All authors read and approved the final manuscript.

## Pre-publication history

The pre-publication history for this paper can be accessed here:

http://www.biomedcentral.com/1471-2407/11/374/prepub

## Supplementary Material

Additional file 1 **AUC values and corresponding sensitivity and specificity of single miRNAs for distinguishing lung cancer from benign SPNs**. AUC, the area under receiver operating characteristic curve. Click here for file

Additional file 2 **ROC curves analysis of expression levels of plasma miRNAs**. ROC curves analysis of expression levels of plasma miRNAs in 32 patients with malignant SPNs and 33 patients with benign SPNs. The AUC conveys accuracy miRNA in distinguishing malignant from benign SPNs in terms of sensitivity and specificity. A-E show ROC curves of five individual miRNAs, respectively. F shows ROC curve of combined three miRNAs, miR-21, miR-210, and miR-486-5p, a composite panel. Click here for file

Additional file 3 **Pearson correlation analysis of coefficients between the three miRNAs**. Coefficients between the three miRNAs were determined by using Pearson Correlation analysis. Click here for file

Additional file 4 **Relationship between plasma miRNA expressions and clinical characteristics**. Relationship between plasma miRNA expressions and demographic and clinical characteristics of the patients and control individuals was analysed by Spearman rank correlation. Click here for file

Additional file 5 **Relationship between the three miRNAs and clinical characteristics**. Relationship between the three miRNAs and demographic and clinical characteristics of the patients with malignant SPNs and patients with benign SPNs was determined by Spearman rank correlation. Click here for file
