# Supplementary material for: Diagnosis of lung cancer in individuals with solitary pulmonary nodules by plasma microRNA biomarkers
Source: BMC Cancer. 2011 Aug 24;11:374. doi: 10.1186/1471-2407-11-374 (PMC3175224; doi:10.1186/1471-2407-11-374)
Supplement: Additional file 1 — AUC values and corresponding sensitivity and specificity of single miRNAs for distinguishing lung cancer from benign SPNs. AUC, the area under receiver operating characteristic curve. [file 1471-2407-11-374-S1.DOCX]

| **Table S1.** AUC values and corresponding sensitivity and specificity of single miRNAs for distinguishing lung cancer from benign SPNs | | | |
| --- | --- | --- | --- |
| miRNAs | AUC (95% CI) | Sensitivity (95% CI) | Specificity (95% CI) |
| miR-375 | 0.6088 (0.4721 to 0.7455) | 66.7% (48.17% to 82.04%) | 57.58% (39.22% to 74.52%) |
| miR-126 | 0.5767 (0.4348 to 0.7186) | 62.5% (43.69% to 78.90%) | 63.64% (45.12% to 79.60%) |
| miR-21 | 0.5862 (0.4348 to 0.7186) | 56.25% (37.66% to 73.64%) | 63.64% (45.12% to 79.60%) |
| miR-210 | 0.6913 (0.5611 to 0.8215) | 56.25% (37.66% to 73.64%) | 72.73% (54.48% to 86.70%) |
| miR-486-5p | 0.6288 (0.5779 to 0.8312) | 71.8% (53.25% to 86.25%) | 66.67% (48.17% to 82.04%) |
| AUC, the area under receiver operating characteristic curve. | | |  |
