# Supplementary figures and images for: Diagnosis of lung cancer in individuals with solitary pulmonary nodules by plasma microRNA biomarkers
Source: BMC Cancer. 2011 Aug 24;11:374. doi: 10.1186/1471-2407-11-374 (PMC3175224; doi:10.1186/1471-2407-11-374)

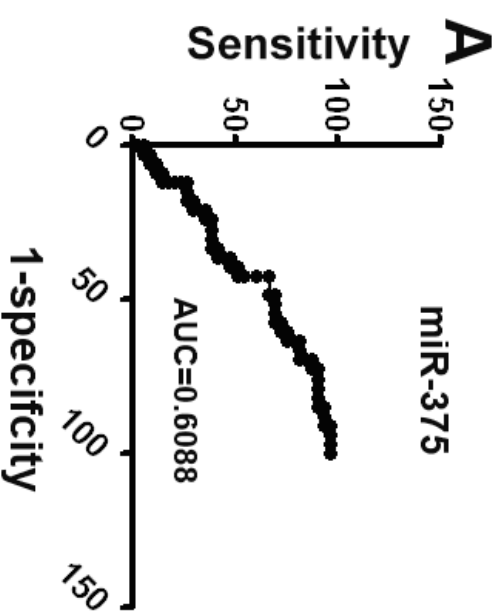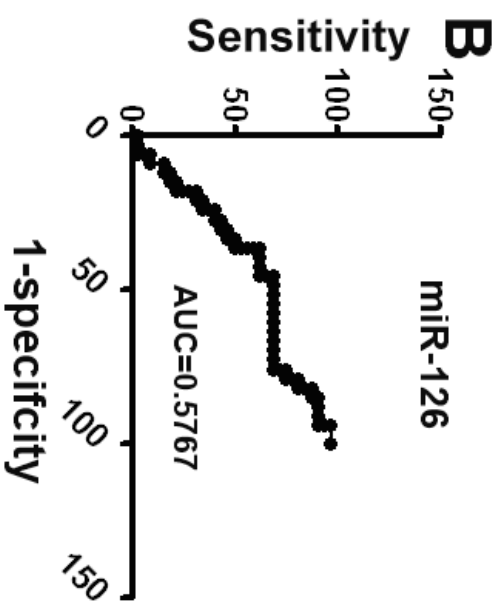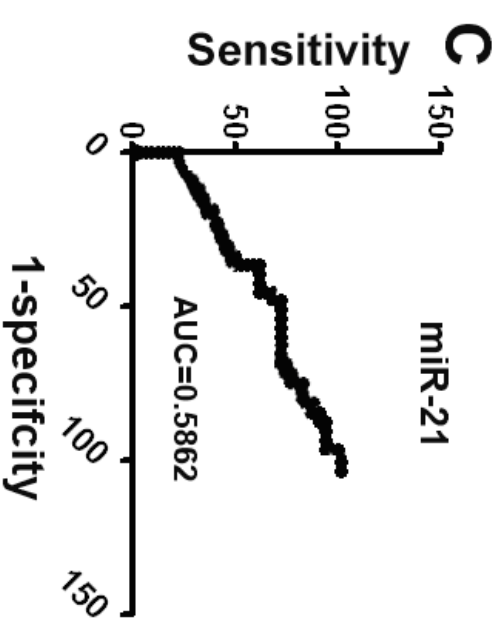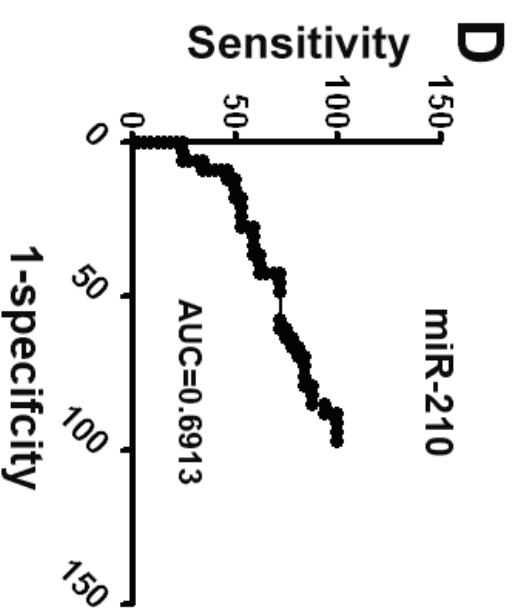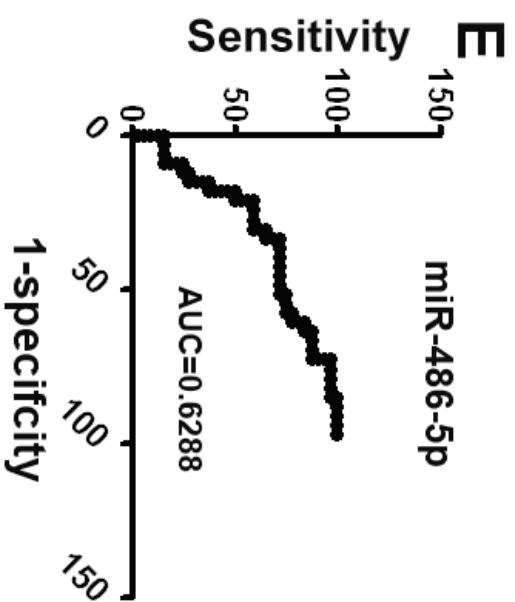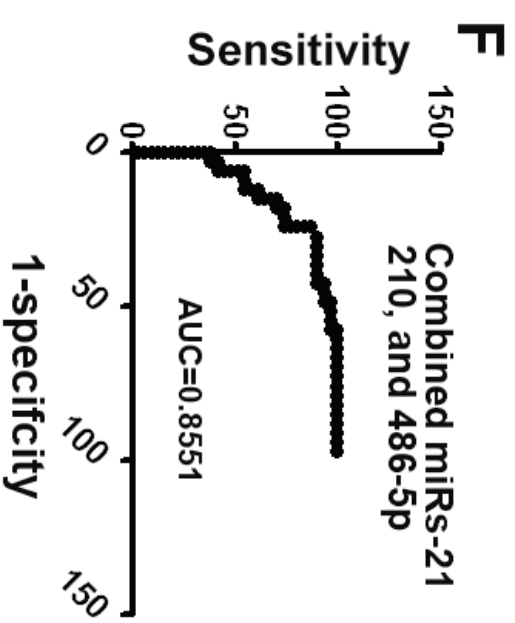

Supplement: Additional file 2 — ROC curves analysis of expression levels of plasma miRNAs. ROC curves analysis of expression levels of plasma miRNAs in 32 patients with malignant SPNs and 33 patients with benign SPNs. The AUC conveys accuracy miRNA in distinguishing malignant from benign SPNs in terms of sensitivity and specificity. A-E show ROC curves of five individual miRNAs, respectively. F shows ROC curve of combined three miRNAs, miR-21, miR-210, and miR-486-5p, a composite panel. [file 1471-2407-11-374-S2.PDF]
