# Supplementary material for: Diagnosis of lung cancer in individuals with solitary pulmonary nodules by plasma microRNA biomarkers
Source: BMC Cancer. 2011 Aug 24;11:374. doi: 10.1186/1471-2407-11-374 (PMC3175224; doi:10.1186/1471-2407-11-374)
Supplement: Additional file 3 — Pearson correlation analysis of coefficients between the three miRNAs. Coefficients between the three miRNAs were determined by using Pearson Correlation analysis. [file 1471-2407-11-374-S3.DOCX]

| **Table S2.** Pearson correlation analysis of coefficients between the three miRNAs | | | |
| --- | --- | --- | --- |
|  | miR-21 | miR-210 | miR-486-5p |
| miR-21 | 1.00000 | -0.13772 | 0.09419 |
| miR-210 | 0.29193 | 1.00000 | -0.13566 |
| miR-486-5p | 0.08967 | -0.16786 | 1.00000 |
