# Supplementary material for: Diagnosis of lung cancer in individuals with solitary pulmonary nodules by plasma microRNA biomarkers
Source: BMC Cancer. 2011 Aug 24;11:374. doi: 10.1186/1471-2407-11-374 (PMC3175224; doi:10.1186/1471-2407-11-374)
Supplement: Additional file 4 — Relationship between plasma miRNA expressions and clinical characteristics. Relationship between plasma miRNA expressions and demographic and clinical characteristics of the patients and control individuals was analysed by Spearman rank correlation. [file 1471-2407-11-374-S4.DOCX]

| **Table S3.** **Relationship between plasma miRNA expressions and clinical characteristics** | | | | | | | |
| --- | --- | --- | --- | --- | --- | --- | --- |
| MiRNAs | Age | Sex | Race | Smoking Pack-years | Nodule size | Stage | Histological types |
| miR-375 | 0.065 (0.541) | -0.099 (0.402) | 0.021 (0.857) | 0.696 (0.003) * | 0.214 (0.090) | -0.009 (0.93) | -0.228 (0.061) |
| miR-126 | -0.038 (0.725) | -0.044 (0.709) | -0.142 (0.395) | 0.909 (<0.001) * | 0.038 (0.0711) | -0.012 (0.904) | 0.230 (0.06) |
| miR-21 | 0.073 (0.481) | -0.075 (0.528) | -0.119 (0.314) | 0.26 (0.008) * | 0.090 (0.0384) * | -0.149 (0.135) | 0.010 (0.931) |
| miR-210 | -0.042 (0.688) | -0.202 (0.085) | 0.123 (0.295) | 0.89 (0.006) * | 0.213 (0.0154) * | -0.133 (0.182) | -0.052 (0.657) |
| miR-486-5p | 0.159 (0.304) | -0.138 (0.277) | -0.028 (0.825) | 0.28 (0.003) * | 0.136 (0.0219) * | -0.121 (0.411) | -0.046 (0.784) |
| Relationship between plasma miRNA expressions and demographic and clinical characteristics of the patients and control individuals was analyzed by Spearman rank correlation.  *, the demographic and clinical characteristics that are statistically associated with plasma miRNA expressions (P value of <0.05). | | | | | | | |
