# Supplementary material for: Diagnosis of lung cancer in individuals with solitary pulmonary nodules by plasma microRNA biomarkers
Source: BMC Cancer. 2011 Aug 24;11:374. doi: 10.1186/1471-2407-11-374 (PMC3175224; doi:10.1186/1471-2407-11-374)
Supplement: Additional file 5 — Relationship between the three miRNAs and clinical characteristics. Relationship between the three miRNAs and demographic and clinical characteristics of the patients with malignant SPNs and patients with benign SPNs was determined by Spearman rank correlation. [file 1471-2407-11-374-S5.DOCX]

| **Table S4. Relationship between the three miRNAs and clinical characteristics** | | | | | | |
| --- | --- | --- | --- | --- | --- | --- |
| MiRNAs | Age | Sex | Smoking Pack-years | Nodule size | Stage | Histological types |
| miR-21 | 0.098 (0.367) | 0.126 (0.023) | 0.21 (0.003) * | 0.07 (0.023) * | 0.08 (0.067) | 0.02 (0.678) |
| miR-210 | 0.021 (0.254) | 0.237 (0.231) | 0.80 (0.030) * | 0.04 (0.0136) * | 0.031 (0.065) | 0.0432 (0.756) |
| miR-486-5p | 0.178 (0.456) | 0.238 (0.476) | 0.267 (0.021) * | 0.187 (0.012) * | 0.156 (0.113) | 0.098 (0.767) |
| Relationship between the three miRNAs and demographic and clinical characteristics of the patients with malignant SPNs and patients with benign SPNs was determined by Spearman rank correlation.  *, a P value of <0.05 was considered statistically significant. | | | | | | |
